# The depressive disturbances characteristic of forsed migrants during military conditions

**DOI:** 10.1192/j.eurpsy.2026.11407

**Published:** 2026-07-31

**Authors:** B. Mykhaylov

**Affiliations:** Shupyk National Healthcare University of Ukraine, Kyiv, Ukraine

## Abstract

**Introduction:**

Forced displacement due to armed conflict is a significant risk factor for the development of various mental health disorders. Among the most prevalent are depressive disorders, which vary in severity and clinical presentation. Understanding their phenomenology in displaced populations is critical for effective diagnosis and intervention.

**Objectives:**

To compare the clinical features of depressive disorders in forcibly displaced persons, specifically distinguishing between recurrent depressive disorder (RDD, F33) and prolonged depressive reaction (PDR, F43.21) as a form of adjustment disorder.

**Methods:**

A total of 96 internally displaced persons (IDPs) with depressive symptoms were examined. Participants were divided into two groups: 34 with signs of RDD and 62 with signs of PDR. The study utilized clinical-psychopathological interviews and psychodiagnostic tools: the Hamilton Depression Rating Scale (HDRS) and the Beck Depression Inventory (BDI).

**Results:**

The PDR group primarily exhibited somatic symptoms (sleep disturbances, reduced appetite, decreased libido and work capacity), with less pronounced affective symptoms. The RDD group demonstrated both somatic and affective-cognitive symptoms, including guilt and suicidal ideation.

Psychodiagnostic results showed:

- PDR group: 81.08% had mild depressive symptoms, 17.57% had none.
- RDD group: 62.5% had mild, 37.5% moderate symptoms.

According to BDI, PDR was characterized by somatic complaints, while RDD showed dominant affective-cognitive manifestations. Self-assessments confirmed greater emotional burden in the RDD group.

**Conclusions:**

Displaced persons with RDD exhibit more severe and cognitively loaded depressive symptoms than those with PDR, who primarily present with somatic complaints. These findings highlight the need for differentiated diagnostic and therapeutic approaches tailored to the specific subtype of depressive disturbance.

**Disclosure of Interest:**

None Declared

